# Maternal embryonic leucine zipper kinase regulates pancreatic ductal, but not *β*‐cell, regeneration

**DOI:** 10.14814/phy2.12131

**Published:** 2014-09-04

**Authors:** Cheng‐Ho Chung, Amber Miller, Andreas Panopoulos, Ergeng Hao, Robert Margolis, Alexey Terskikh, Fred Levine

**Affiliations:** 1 Mackay Memorial Hospital, Taipei, Taiwan; 2 Sanford‐Burnham Medical Research Institute, La Jolla, California

**Keywords:** Cancer, duct, MELK, migration

## Abstract

The maternal embryonic leucine zipper kinase (MELK) is expressed in stem/progenitor cells in some adult tissues, where it has been implicated in diverse biological processes, including the control of cell proliferation. Here, we described studies on its role in adult pancreatic regeneration in response to injury induced by duct ligation and *β*‐cell ablation. MELK expression was studied using transgenic mice expressing GFP under the control of the MELK promoter, and the role of MELK was studied using transgenic mice deleted in the MELK kinase domain. Pancreatic damage was initiated using duct ligation and chemical beta‐cell ablation. By tracing MELK expression using a MELK promoter‐GFP transgene, we determined that expression was extremely low in the normal pancreas. However, following duct ligation and *β*‐cell ablation, it became highly expressed in pancreatic ductal cells while remaining weakly expressed in *α*‐cells and *β*‐ cells. In a mutant mouse in which the MELK kinase domain was deleted, there was no effect on pancreatic development. There was no apparent effect on islet regeneration, either. However, following duct ligation there was a dramatic increase in the number of small ducts, but no change in the total number of duct cells or duct cell proliferation. In vitro studies indicated that this was likely due to a defect in cell migration. These results implicate MELK in the control of the response of the pancreas to injury, specifically controlling cell migration in normal and transformed pancreatic duct cells.

## Abstract (precis)

The manuscript reports the discovery of a previously unrecognized role for the MELK kinase in regulating pancreatic duct cell migration, which plays an important role in the response of the pancreas to injury.

## Introduction

The maternal embryonic leucine zipper kinase (MELK) is a member of the Snf1/AMPK kinase family (Heyer et al. 1997). While its function remains obscure, it has been implicated in cell cycle regulation (Davezac et al. 2002), cell proliferation (Nakano et al. 2005), pre‐mRNA processing (Vulsteke et al. 2004), cell division, and cytokinesis (Le Page et al. 2011). In adult mice, MELK is enriched in the ovary, testis, lung, and thymus (Heyer et al. 1999). It appears to be expressed in several stem/progenitor cell populations in adult animals, including hematopoietic stem cells (Easterday et al. 2003), and multipotent neural progenitors (Nakano et al. 2005). MELK expression is also elevated in a variety of cancers (Hemmati et al. 2003; Rhodes et al. 2004) and is associated with poor patient survival in breast cancer and astrocytoma (Marie et al. 2008; Pickard et al. 2009). However, little is known about the functional significance of elevated MELK expression in cancer, its role in stem/progenitor cells, or indeed whether it plays a role in development or adult organ homeostasis.

**Figure 1.**
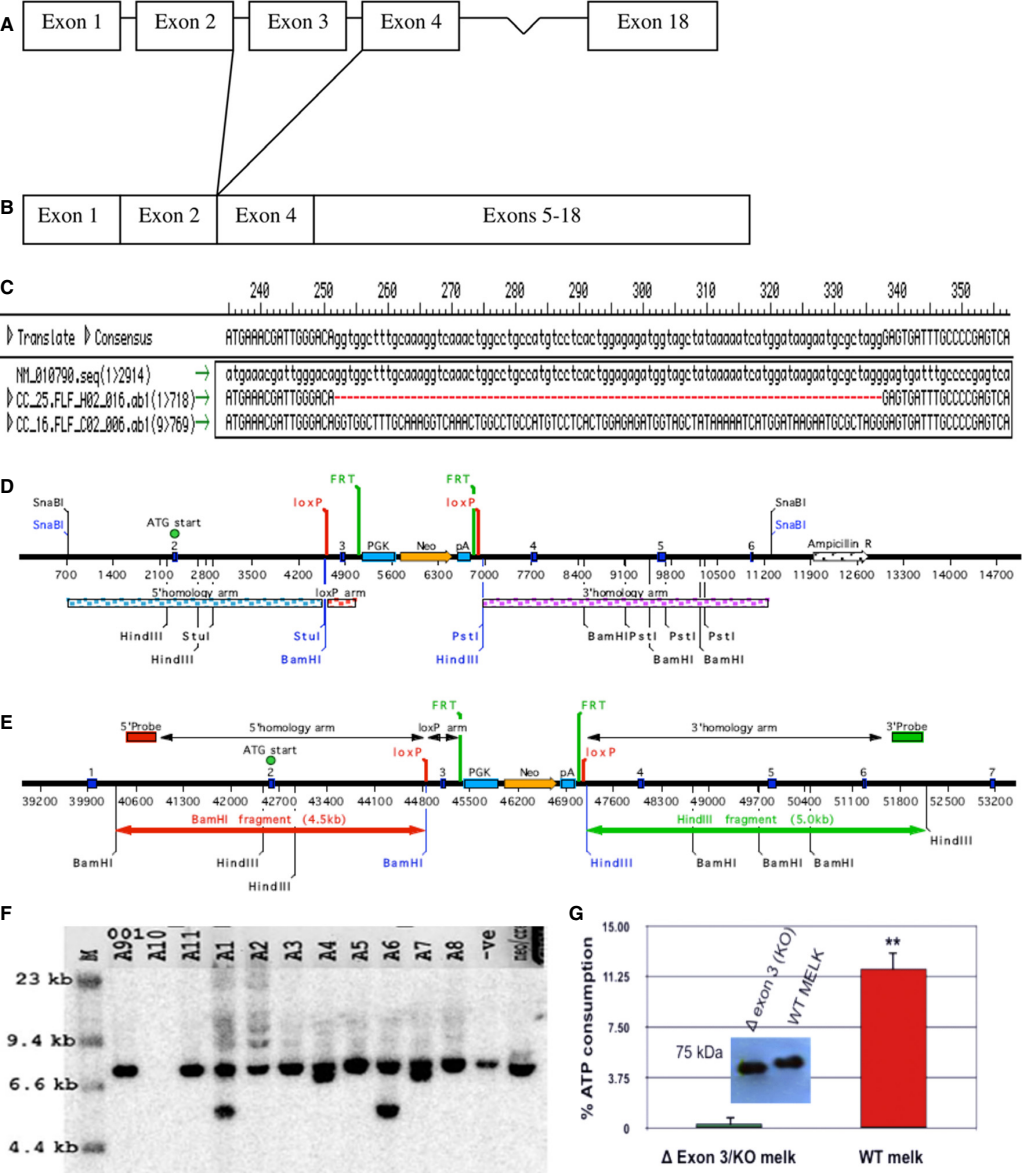
Schematics and sequencing of MELK wild type and Delta 3 MELK cDNA (A–C) and the targeting scheme for delta 3 MELK construction (D–F). (A) Schematic of MELK exons 1–18. (B) Schematic of the exon 3 deletion (Delta 3 MELK) resulting in a frame shift/premature stop codon. (C) Sequence alignment of the PubMed MELK cDNA sequence (NM_010790, first lane), MELK cDNA cloned from the Delta 3 MELK littermate (CC_25.FLF_H02_016, second lane), and MELK cDNA cloned from the WT littermate (OC_16.FLF_CO2_006, third lane). MELK Conditional knockout mice (F1 from Cre/wild type (wt) cross) (D) MELK Flox targeting vector. (E) Targeted genomic locus with restriction enzymes. (F) Southern blot genotyping of MELK Flox and Delta 3 MELK mice. DNA was digested with StuI and probed with the 3′ probe. These mice are a result of chimera x Oz‐Cre deleter mating. The expected band sizes are: wt 7.3 kb, flox 6.8 kb, and Delta 3 MELK 5.3 kb. A1 and A6 are wt/Delta 3 MELK/Cre. A4 and A7 are wt/flox. (G) Verification of the effect of kinase domain deletion on MELK kinase activity. The MELK cDNA lacking the 3d exon was recovered from the ∆3MELK mice by RT‐PCR and engineered with an alternative in‐frame ATG start codon to allow protein expression. The C‐termimnal FLAG‐tag was added for affinity purification and Western blot detection (inset). The Kinase‐Glo Plus Luminescent Kinase Assay (Promega) was used to measure kinase activity using MBP as a generic substrate. The mutant ∆3MELK protein showed no detectable kinase activity compared to the wild‐type recombinant MELK protein.

To study the role of MELK, we used two transgenic mouse strains: a previously described mouse in which eGFP is driven by a MELK promoter fragment (Nakano et al. 2005; Hebbard et al. 2010), and a newly developed mouse in which one exon encoding the MELK kinase domain was deleted (Delta 3 MELK mice) (Fig. 1). MELK kinase domain deletion had no apparent effect, as homozygous mutant mice were born in normal Mendelian ratios with no obvious effect on health.

Given the expression of MELK in stem/progenitor cells and the lack of an obvious effect of deleting the MELK domain, we decided to investigate a possible role for MELK in response to injury. Thus, we studied a model of pancreatic damage combining pancreatic duct ligation (PDL) and *β*‐cell ablation by alloxan. In that model, combining *β*‐cell ablation by alloxan with the complex effects of PDL that includes loss of acinar tissue and ductal cell hyperplasia (Chung et al. 2010), we previously demonstrated efficient *β*‐cell neogenesis by direct conversion from preexisting *α*‐cells (Chung et al. 2010). While the lack of MELK kinase function had no effect on adult *β*‐cell regeneration from *α*‐cells, we did find that MELK regulated the size and number of regenerative ducts. This phenomenon may be through an effect of MELK on cell migration, as we found that downregulation of MELK by siRNA had a profound effect on cell migration in vitro.

## Material and Methods

### Animal and animal procedures

MELK‐GFP mice have been described previously (Nakano et al. 2005). They were generated by lentiviral‐mediated integration of the 3.5 kB MELK proximal promoter driving GFP into oocytes injected with lentiviral vectors. In these transgenic mice, the GFP expression recapitulated the expression pattern of endogenous MELK mRNA, being largely restricted to developing GZ, including the GZ surrounding the lateral ventricles and the rostral migratory stream, the inner granule zone of the early postnatal hippocampus, and external granule cells of the neonatal cerebellum (Nakano et al. 2005).

To generate Delta 3 MELK mice, the 3rd exon in the MELK gene was flanked with loxP sites to allow Cre‐mediated recombination (Fig. 1D, E and F). The Delta 3 MELK mice were obtained by mating MELK flox mice with ubiquitously expressed Cre (OZ‐Cre) deleter strain (generation of targeted ES cell and mouse lines was done by Ozgene Inc., Perth, Australia). Splicing of exon 2 to exon 4 results in a frameshift mutation with the introduction of a premature TGA stop codon. However, due to aberrant translational initiation from a cryptic ATG codon in the 2^nd^ exon, naturally occurring in breast cancers, smaller 71 and 66 kDa kinase‐dead variants of MELK could be generated in these mice. These MELK variants lack kinase activity due to the deletion of critical residues (3rd or 3rd and 4th exons) in the kinase catalytic domain. We also generated Delta 3 MELK‐GFP mice by breeding Delta 3 MELK with MELK‐GFP mice.

All animal experiments were approved by the Institutional Animal Care and Use Committee of the Sanford‐Burnham Medical Research Institute and in accordance with national regulations. Eight‐ to twelve‐week‐old C57/B6 mice (Harlan Sprague Dawley, Inc., Placentia, CA), MELK‐GFP mice, Delta 3 MELK mice, and Delta 3 MELK‐GFP mice were injected intravenously with Alloxan (Sigma Aldrich, St. Louis, MO) at 90 mg/kg. PDL was performed 30 min after alloxan injection as described (Chung et al. 2010). Diabetic mice were injected subcutaneously once daily with insulin glargine (Sanofi‐Aventis, Paris, France) as described (Chung et al. 2010).

### Immunohistochemical staining

Tissue was fixed in 4% formaldehyde for 6 h at 4°C, washed in PBS (Phosphate buffered saline), followed by overnight in 30% sucrose at 4°C, then embedded in OCT (Optimal Cutting Temperature) compound and frozen at 80°C. Cryosections of 5 *μ*m thickness were incubated with antisera specific for insulin (1/200, guinea pig, USBIO, Swampscott, MA), glucagon (1/50, rabbit, Abcam, Cambridge, MA), cytokeratin (Wide Spectrum Screening; 1/500, rabbit, DakoCytomation, Glostrup, Denmark), KI67 (1/50, mouse, BD Pharmingen, San Diego, CA), green fluorescent protein (GFP) (1/500, chicken, Abcam). Secondary antibodies for detection of guinea pig, rabbit, or mouse antibodies were labeled with: Alexa Fluor 488 (Invitrogen, Carlsbad, CA), Rhodamine Red (Jackson ImmunoResearch Laboratories, West Grove, PA), Cy5 (Jackson ImmunoResearch Laboratories). Nuclei were visualized with DAPI (40, 6‐diamidino‐2‐phenylindole) (Sigma Aldrich).

### Cell culture

Human pancreatic carcinoma Panc‐1 cells (ATCC CRL‐1469, Manassas, VA) were cultured in DMEM (Gibco) with 10% fetal bovine serum, 100 U/mL penicillin, and 100 mg/mL streptomycin.

To generate Panc‐1 clones, Panc‐1 cells were dissociated with 0.05% trypsin (GIBCO), washed once, and then resuspended in cold PBS containing 0.5% FBS. Single Panc‐1 cells were inoculated into 96 wells using a BD FACSDiVa 7‐color high‐speed cell sorter (BD Biosciences, San Jose, CA). Single cell seeding was confirmed using a light microscope 6–8 h later.

### Quantification of *α*‐cell, *β*‐cell, and duct area

For the quantitative analysis of glucagon, (*α*‐cell) insulin (*β*‐cell), and pancytokeratin (duct) area, we studied sections spaced 100 *μ*m aside from each other from the tail of the pancreas per mouse.

These sections were incubated with antisera to insulin, glucagon, and pancytokeratin as previously described (Chung et al. 2010). The nuclei were stained by DAPI. All slides were scanned using the Aperio ScanScope XT system) (version 10, Aperio Technologies, Vista, CA) and quantification of cells and area was done using an automated image analysis algorithm through CyteSeer software (Vala Sciences, San Diego, CA.

### Lentiviral infection

Panc‐1 clones were infected with previously described lentiviral constructs on the SIN18 backbone expressing either MELK shRNA (directed against the 3′ untranslated region of the endogenous MELK transcript) from the U6 promoter and nuclear mCherry from the PGK promoter, or scrambled‐MELK‐shRNA and nuclear mCherry as a control (Hebbard et al. 2010). The specificity of the effect of the MELK shRNA was demonstrated by transfection of a MELK cDNA, leading to reversal of biological effects of the shRNA (Hebbard et al. 2010). Multiplicity of infection was ~50.

### RNA isolation and analysis

RNA extracts were prepared using the Qiagen RNeasy Mini Kit and the on‐column RNase‐free DNase set (Qiagen, Germantown, MD). cDNA was synthesized using the Superscript III First‐Strand Synthesis System (Invitrogen). Quantitative PCR was performed using the Taqman gene expression primer/probe TAMRA sets and an ABI PRISM 7700 Sequence Detection System (Applied Biosystems, Foster City, CA). All Ct data were normalized to the 18S rRNA‐internal control and delta *C*_*t*_ values were calculated.

### Scratch assay

For the evaluation of Panc‐1 motility in vitro, a wounding (scratch) assay was performed. Panc‐1 cells infected with lentiviruses expressing either MELK shRNA and nuclear mCherry or scrambled‐MELK‐shRNA and nuclear mCherry, were allowed to grow to confluent status and a linear scratch wound was made with a 10 *μ*L pipet tip, after which the cells were cultured for 21 h. To evaluate directional cell migration, time‐lapse images were acquired during the 21 h at 10 min intervals.

### Single cell migration assay

Panc‐1 cells transfected with scrambled shRNA or MELK shRNA were separated into single cells and then seeded in 96‐well cell culture plates. Time‐lapse images were acquired for 12 h at 20 min intervals to evaluate random cell migration.

### Image and statistical analysis

Confocal images were acquired by Radiance 2100/AGR‐3Q BioRad Multi‐photon Laser Point Scanning Confocal Microscope. Statistical significance of changes in controls versus experimental groups was calculated by an unpaired Student's *t*‐test. We considered *P*‐values below 0.05 as statistically significant. For all statistical analysis, Graphpad Prism 5 (GraphPad Software, La Jolla, CA) was used. All results are expressed as mean ± SD.

## Results

### MELK kinase domain deletion had no effect on pancreatic morphology or function

**Figure 2.**
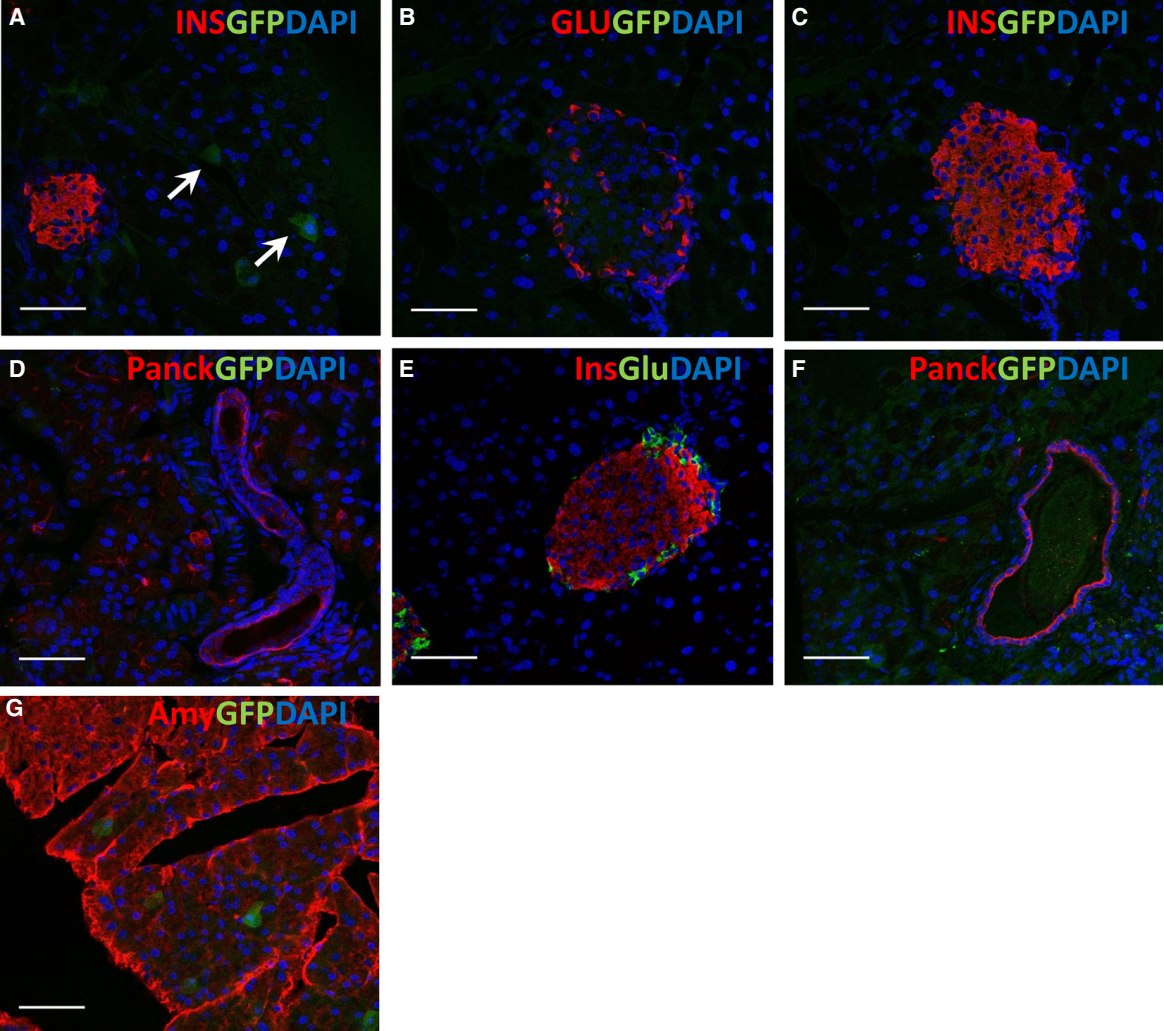
MELK expression in normal adult mouse pancreas. Representative sections from the pancreas of a normal MELK‐GFP mouse (A–D, G) and a Delta 3 MELK‐GFP mouse (E, F). Sections were immunostained with the indicated antibodies (Ins: Insulin, Glu: Glucagon, Amy: Amylase, Panck: Pancytokeratin). Nuclei were visualized with DAPI (blue). GFP driven by the MELK promoter‐GFP transgene is green in (A–D, F, G). GFP expression was weak and was seen only in a subset of acinar cells in the normal adult pancreas (A, G). *α*‐cells, *β*‐cells, and ductal cells in normal adult pancreas did not express MELK (B, C, D). Mice without MELK kinase function had normal islet and ductal morphology (E, F). Scale bar = 50 *μ*m.

MELK expression was analyzed using adult MELK‐GFP mice (Nakano et al. 2005) because reliable MELK antibodies are not available. In the pancreas, MELK‐GFP was weakly expressed in a subset of adult acinar cells (Fig. 2A and G), but not in islet or ductal cells (Fig. 2B, C and D). Adult Delta 3 MELK mice exhibited normal islet and duct morphology (Fig. 2E and F) and were euglycemic (not shown).

### MELK expression was induced in *α*‐cells, *β*‐cells, and ductal cells following injury to the adult pancreas

**Figure 3.**
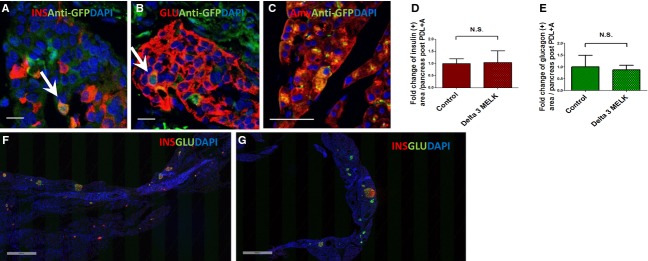
The effect of MELK functional deficiency on adult *β*‐cell neogenesis from mature *α*‐cells. Representative sections from the ligated part (A, B) and unligated part (C) of MELK‐GFP mouse pancreas 14 days after PDL plus alloxan were examined. Sections were immunostained with the indicated antibodies (Ins: Insulin, Glu: Glucagon, Amy: Amylase, Anti‐GFP: GFP antibody). Nuclei were visualized with DAPI (blue). PDL plus alloxan induced weak GFP expression in *β*‐ (A) and *α*‐cells (B). However, the lack of MELK kinase function did not significantly affect the number of regenerating *β*‐cells (D) or the total number of *α*‐cells (E) after PDL plus alloxan (*n* = 3 in control and Delta 3 MELK group). Scale bar = 10 *μ*m (A, B). Panel C showed that acinar cells in the unligated part of pancreas have GFP expression similar to the GFP expression in *β*‐ (A) and *α*‐cells (B). Scale bar = 50 *μ*m. (F, G) Representative additional adult mouse pancreatic sections taken at low magnification through the entire pancreas from MELK‐GFP mice (F) or delta 3 MELK‐GFP mice (G) 14 days after PDL plus alloxan demonstrated that the loss of MELK kinase function did not interfere with *β*‐cell formation following PDL plus alloxan treatment. Slides were costained with insulin (red), glucagon (green), and DAPI (blue). Arrows indicate cells coexpressing the indicated hormone plus MELK, thus having a yellowish color. Scale bar = 500 *μ*m (F, G).

Since the deletion of the MELK kinase domain had no effect on pancreatic development, we evaluated its role in regeneration in the mature pancreas. To that end, we employed a new damage model combining PDL**,** plus alloxan injection described previously (Chung et al. 2010). Briefly, the pancreatic duct was ligated midway between the head and tail of the organ (Wang et al. 1995). This led to disappearance of acinar markers and to marked ductal hyperplasia (Xu et al. 2008). Following PDL plus alloxan, MELK, which in the normal pancreas was only weakly expressed in a subset of acinar cells, became weakly expressed in a subset of *β*‐cells (Fig. 3A) and *α*‐cells (Fig. 2B and B) and was highly expressed in duct cells (Fig. 3A and B). There was no change in MELK expression proximal to the ligation, with some acinar cells retaining weak GFP expression (Fig. 3C).

### MELK regulated the size and number of regenerative ducts in adult pancreas after injury

**Figure 4.**
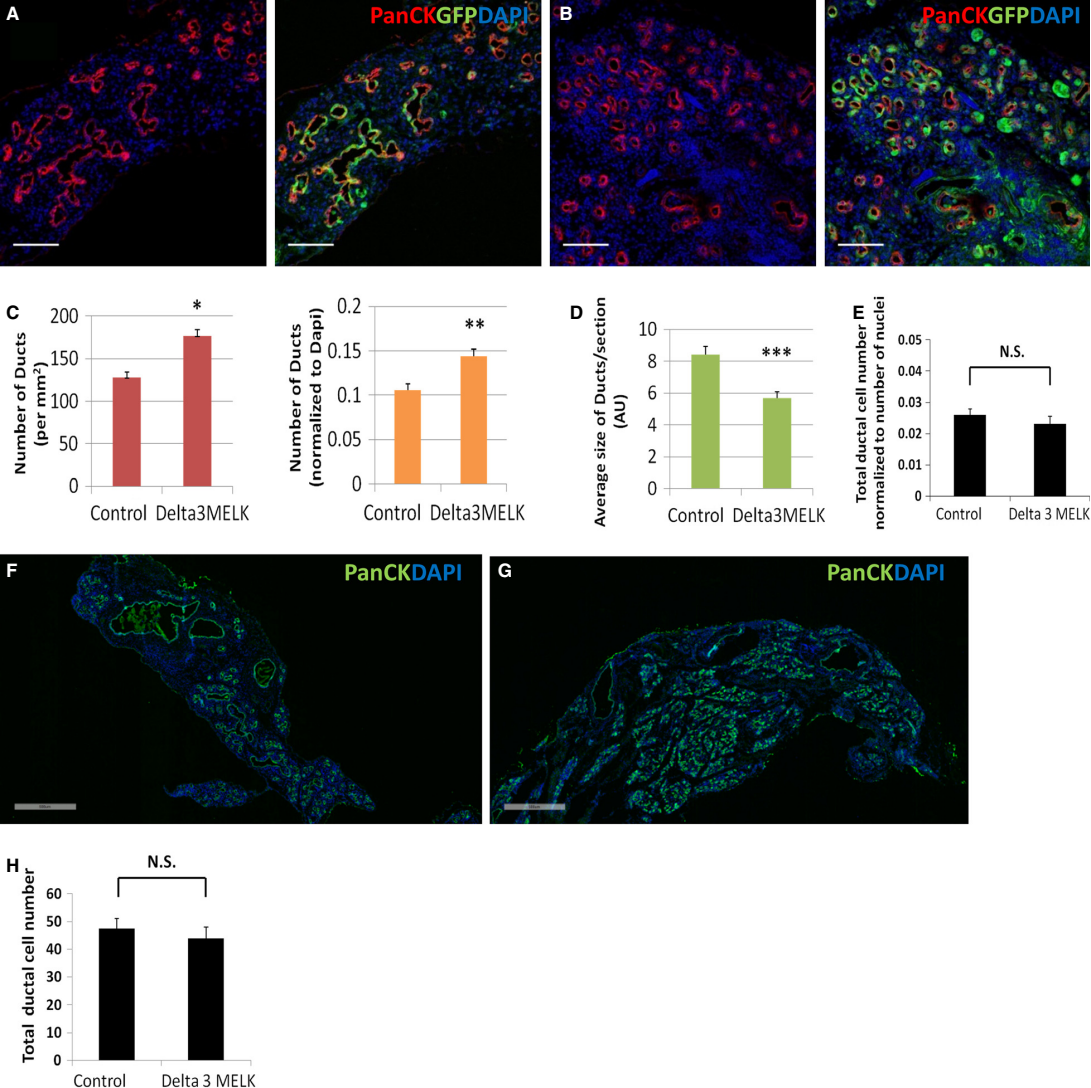
The effect of MELK functional deficiency on adult pancreatic ductal regeneration. Representative adult mouse pancreatic sections from MELK‐GFP mice (A) or Delta 3 MELK‐GFP mice (B) 14 days after PDL plus alloxan analyzed with an antibody to all cytokeratins (PanCK, red). Endogenous GFP detects the MELK‐GFP transgene and DAPI detects nuclei. PDL plus alloxan strongly induced MELK expression in adult pancreatic ductal cells (A, B). Functional deficiency of MELK resulted in smaller size duct formation during adult pancreatic duct regeneration (A,B, quantified in D). However, the density of ducts become higher (A, B, quantified in C). Data in C were normalized to pancreatic area (left panel) or to the number of nuclei (right panel). There was no difference in the number of ductal cells, either when normalized to the number of nuclei (E) or not normalized (H). Scale bar = 100 *μ*m. Additional representative adult mouse pancreatic sections with low magnification from MELK‐GFP mice (F) or delta 3 MELK‐GFP mice (G) 14 days after PDL plus alloxan demonstrated the changed ductal size and ductal number in the delta 3 MELK‐GFP mouse following PDL plus alloxan. Slides were costained with anti‐pancytokeratin (Panck, green), and DAPI (blue). Scale bar, 500 *μ*m.

**Figure 5.**
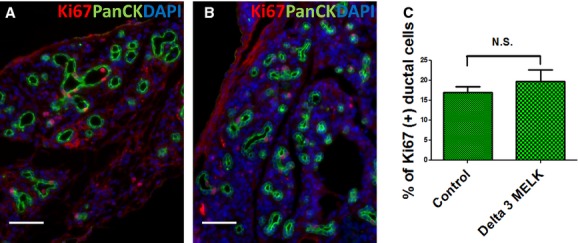
MELK kinase domain deletion did not affect adult pancreatic ductal replication. Representative adult mouse pancreatic sections from control (A) or delta 3 MELK mice (B) 14 days after PDL plus alloxan analyzed by immunofluorescence microscopy for Ki67 (red), pancytokeratin (green), and DAPI (blue). There was no difference in the number of Ki67‐positive cells between control and delta 3 MELK mice (C) (*n* = 3 in control and Delta 3 MELK group). Scale bar = 100 *μ*m.

MELK kinase domain deletion had a dramatic effect on the morphology of ducts distal to the ligation, but no effect on islet cells. Comparing the mice with loss of MELK kinase function (Fig. 4B and G) to mice with intact MELK kinase (Fig. 4A and F), there was increased ductal density and a decrease in the average size of each duct (Fig. 4C and D), but the total number of duct cells did not change (Fig. 4E). While previous studies had implicated MELK in cell proliferation (Nakano et al. 2005, 2008), we found no difference between Delta 3 MELK mice and control mice in ductal cell replication following PDL plus alloxan (Fig. 5), despite the significant effect of MELK kinase deletion on duct cell morphology (Fig. 4). MELK kinase domain deletion did not significantly affect the number of *β*‐ or *α*‐cells following PDL plus alloxan (Fig. 3D, E, F and G).

### MELK deficiency impaired directional cell migration in vitro

**Figure 6.**
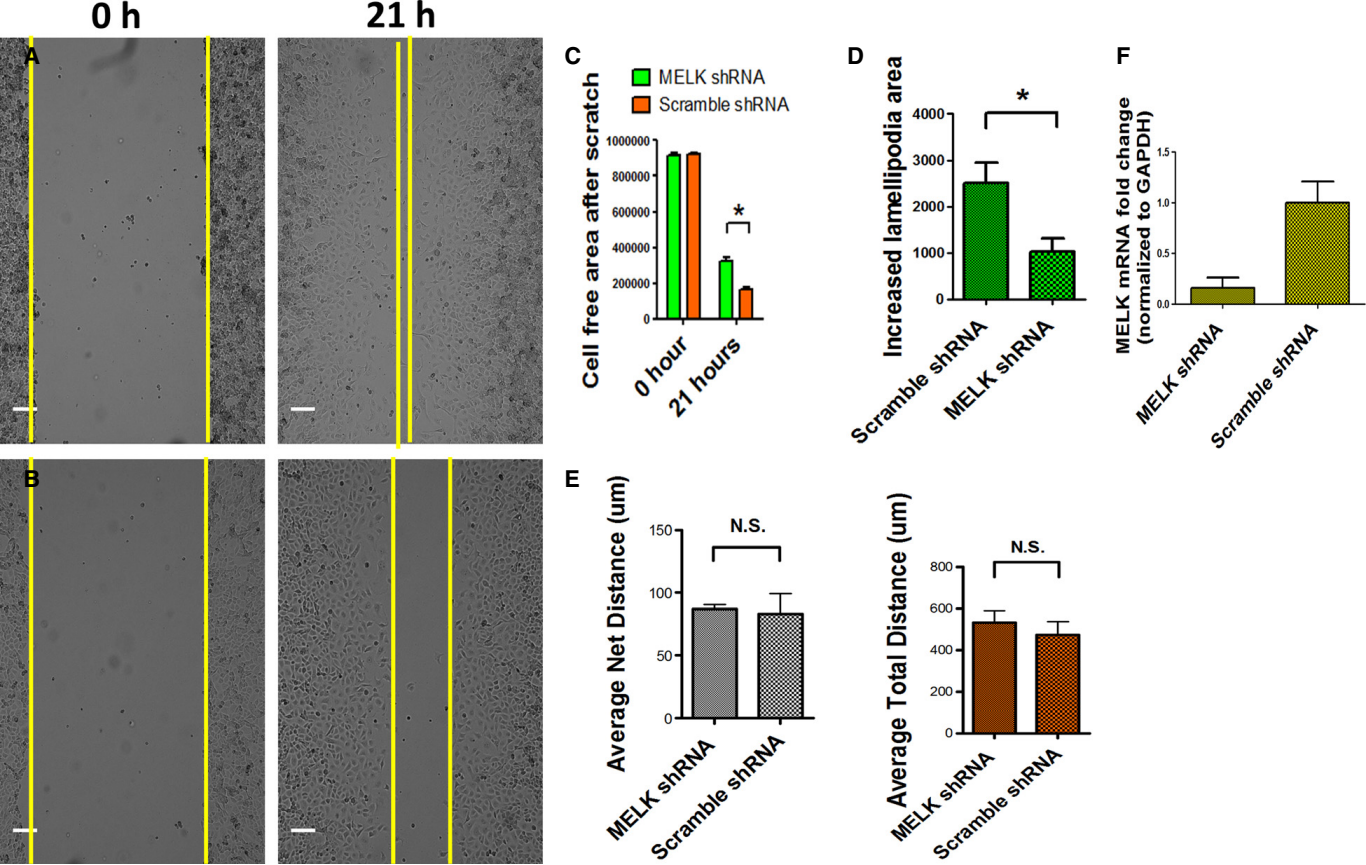
MELK is required for efficient directional cell migration. Phase contrast microscopy of PANC‐1 cells immediately (0 h) and 21 h (A, B) after scratch. The cells were treated with lentiviral vectors expressing either scrambled shRNA (A) or MELK shRNA (B). The MELK shRNA treatment resulted in an 85% reduction of MELK mRNA (F). In a scratch assay, MELK knockdown cells exhibited reduced migration (A, B, quantified in C). Time‐lapse images were also acquired during scratch assay for both MELK shRNA treated and scrambled shRNA treated groups (Supplemental Movie S1, S2). Cells at the edge of the scratch had defective lamellipodia extension in the MELK shRNA condition (D, also see Movie S1, S2). While MELK shRNA had a large effect on directional migration, analysis of the migration of single cells revealed no effect of MELK shRNA on random cell migration‐ either total distance traveled or the net distance of random cell migration (E).

The altered duct morphology and the absence of effect of MELK kinase domain deletion on replication suggested that MELK might be playing a role in cell migration. MELK is overexpressed in many cancer cell types, but it has been studied primarily as a regulator of cellular proliferation, not in migration (Lin et al. 2007; Nakano et al. 2008; Bright et al. 2009; Hebbard et al. 2010; Le Page et al. 2011). To study whether MELK plays a role in cellular migration, we used the human pancreatic ductal adenocarcinoma cell line Panc‐1 as a model. It was chosen over other ductal adenocarcinoma cell lines as it has been commonly used as a model of cancer cell motility (Radulovich et al. 2010). Primary pancreatic epithelial cells with a ductal phenotype do not migrate significantly in vitro (Hao et al. 2006). To control for heterogeneity within the Panc‐1 cells, we used flow cytometry to generate Panc‐1 clones. Cells from the same clone were infected with lentiviruses expressing either MELK shRNA (directed against the 3′ untranslated region of the endogenous MELK transcript) and nuclear mCherry, or scrambled‐MELK‐shRNA and nuclear mCherry as a control (Hebbard et al. 2010). Quantitative RT‐PCR analysis of selected clones demonstrated that MELK shRNA led to an 85% reduction in the level of endogenous MELK mRNA compared with control cells from the same clone infected with the scrambled shRNA vector (Fig. 6F). Using paired clones infected with MELK shRNA or control lentiviruses, we tested the effect of MELK knockdown on directional cell migration using a scratch assay (Liang et al. 2007). We observed an inhibition of cell migration in MELK knockdown PANC‐1 cells (Fig. 5A and B), quantified in C (Liang et al. 2007). We observed an inhibition of cell migration in MELK knockdown PANC‐1 cells (Fig. 6A and B, quantified in 6C, Supplemental Movies S1 and S2). Surprisingly, we found that MELK knockdown impaired lamellipodia extension during cell migration (Fig. 6D, Supplemental Movies S1 and S2). Furthermore, the reduction of MELK also impaired the formation of leading cells during cell migration (Supplemental Movies S1, S2). Interestingly, while a reduction in MELK expression impaired directional cell migration, reducing MELK expression had no effect on random cell migration as measured by total or net distance traveled (Fig. 6E).

## Discussion

The studies presented here demonstrate a role for MELK in response to injury in the adult exocrine pancreas. The principal finding was that MELK kinase function is required for the adult pancreas to maintain proper ductal size and number following the injury induced by PDL. The loss of MELK kinase function led to smaller regenerative ducts and a higher ductal density following PDL, with no change in the level of ductal cell replication or of total ductal cell number.

**Figure 7.**
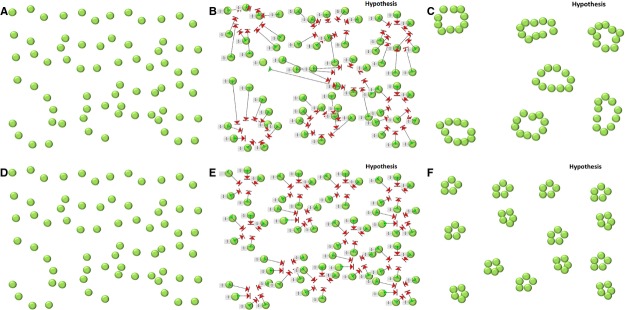
Hypothesis of adult duct regeneration in normal mice (A–C) and Delta 3 MELK mice (D–F). After injury, the structure of the pancreas is severely disrupted and adult pancreatic ductal progenitors (A, D) are guided by extrinsic chemotactic and mechanical cues and migrate coordinately to form cell–cell contacts (B) and subsequent ductal structures (C). Without MELK kinase function (D–F), adult ductal progenitors have impaired directional migration (E), leading to formation of contacts only with neighbors, resulting in a greater number of smaller ducts (F).

After tissue damage, tissue repair requires the recreation of functional multicellular tissue organization, requiring directional and collective cell migration (Friedl and Gilmour 2009; Petrie et al. 2009; Arwert et al. 2012). New ducts and new ductal cells after pancreatic ductal ligation could come from preexisting duct cells (Furuyama et al. 2011) or from acinar cells by acinar to ductal metaplasia (Strobel et al. 2007). In our model, the total number of ductal cells remained unchanged, and there was no effect of MELK kinase mutation on ductal cell replication, indicating that it is unlikely that the loss of MELK kinase function increased the efficiency of acinar to ductal metaplasia. Thus, the alteration in duct morphology appeared to be due to an effect of MELK on cell migration. Adult ductal cells lacking MELK kinase function appeared to have an impaired ability to migrate in response to injury, resulting in smaller ducts with a relatively homogenous size (Figure S5). While our data are suggestive, they do not directly prove a role for MELK in cell migration. However, the findings in vivo were supported by studies with PANC1 cells in vitro, which demonstrated a cell autonomous role for MELK in directional cell migration and leading cell formation (Fig. 7). Additional studies will be required to fully define a role for MELK in cell migration.

Consistent with MELK playing a role predominantly in response to injury, we found that it was barely expressed in the normal pancreas, but was strongly upregulated in ductal cells following injury. This finding echoes previous reports (Easterday et al. 2003; Hemmati et al. 2003; Rhodes et al. 2004; Nakano et al. 2005) which found MELK to be expressed in several adult stem/progenitor populations, including cancer stem cells (Nakano et al. 2005, 2008). However, in contrast to those reports, we found no evidence of a role for MELK in cell proliferation. We also found no role for MELK during development, as there was no apparent effect of MELK kinase deletion on the adult mouse under normal conditions, and the effect on pancreatic ducts was limited to adult regeneration. This could be due to its role being limited to a response to injury in the adult or to functional redundancy during development with other members of the family to which it belongs.

There is increasing evidence that some specific genes are dispensable for embryonic development and normal organ homeostasis, but are essential for adult organ regeneration. One example is focal adhesion kinase, which is dispensable for normal intestinal homeostasis and DNA damage signaling, but is essential for intestinal regeneration (Ashton et al. 2010). These examples challenge the paradigm that adult organ regeneration recapitulates embryonic development.

A caveat with the studies reported here is that the MELK mutation examined was limited to deletion of the kinase domain and may not have completely eliminated MELK expression. Thus, it is possible that the mutated protein retained function other than that involving kinase activity. Regardless, it is clear that MELK plays a role in cell migration, which may be related to its pattern of expression in adult stem/progenitor cells. This finding also has important implications for the role of MELK in cancer, where the focus has been on a role in proliferation. However, the findings presented here suggest that a focus in the future should be on migration.

## Acknowledgments

We are grateful to the SBMRI Animal Facility for the care of the MELK mutant mice and to Alejandro Amador for help with mutant mice and helpful discussions.

## Conflict of Interest

None declared.

## Supplementary Material

Supplementary Movie S1

Supplementary Movie S2
